# New Results for the Error Rate Performance of LoRa Systems over Fading Channels

**DOI:** 10.3390/s22093350

**Published:** 2022-04-27

**Authors:** Kostas Peppas, Spyridon K. Chronopoulos, Dimitrios Loukatos, Konstantinos Arvanitis

**Affiliations:** 1 Department of Informatics and Telecommunications, University of Peloponnese, 22100 Tripoli, Greece; 2 Department of Speech and Language Therapy, University of Ioannina, 45110 Ioannina, Greece; 3 Electronics-Telecommunications and Applications Laboratory, Department of Physics, University of Ioannina, 45110 Ioannina, Greece; 4 Department of Natural Resources Management and Agricultural Engineering, Agricultural University of Athens, 75 Iera Odos Street, Botanikos, 11855 Athens, Greece; dlouka@aua.gr (D.L.); karvan@aua.gr (K.A.)

**Keywords:** bit error rate, fading channels, Internet of things, LoRa, performance evaluation

## Abstract

Long Range (LoRa) systems have recently attracted significant attention within the research community as well as for commercial use due to their ability to transmit data over long distances at a relatively low energy cost. In this study, new results for the bit error rate performance of Long Range (LoRa) systems operating in the presence of Rayleigh, Rice, Nakagami-*m*, Hoyt, η-μ and generalized fading channels are presented. Specifically, we propose novel exact single integral expressions as well as simple, accurate expressions that yield tight results in the entire signal-to-noise ratio (SNR) region. The validity of our newly derived formulas is substantiated by comparing numerically evaluated results with equivalent ones, obtained using Monte-Carlo simulations and exact analytical expressions.

## 1. Introduction

In recent years, the exponential growth in the number of inexpensive, Internet-connected devices has given birth to the Internet of things (IoT) and its numerous applications, including autonomous farming, wearable health monitoring, smart homes and cities. Nevertheless, the increasing number of connected devices in conjunction with memory, bandwidth and energy availability constraints has revealed the limits of traditional connectivity technologies, namely ZigBee, Bluetooth and WiFi, in terms of energy consumption, scalability and throughput [1].

In order to fulfill the communication requirements of the IoT, the so-called Low-Power Wide Area Networks (LPWAN) have recently attracted significant attention within the research community as well as for commercial use, due to their ability to complement traditional cellular and short-range wireless technologies in an efficient manner [2,3,4,5].

Among all available LPWAN protocols, the so-called LoRa (Long Range) technology [6] has emerged as a promising candidate for smart sensing technology for civil (e.g., environment and health monitoring, smart metering, precision agriculture) and industrial applications, in urban and rural environments, due to its long-range and low-power capabilities. LoRa modulation is a 3GPP standard based on the chirp spread-spectrum (CSS) technology [7,8,9,10,11,12,13,14,15,16,17,18,19,20] and uses the industrial, scientific and medical (ISM) frequency bands at 433 MHz, 868 MHz or 915 MHz with data rates of up to 50 kbps.

Although the LoRa technology is well documented in [6], there are still relatively few studies on its theoretical performance. A summary of related works is presented in Table 1.

Specifically, the mathematical representation of the LoRa modulation/demodulation process and its performance in terms of the symbol and bit error rates for additive white Gaussian noise (AWGN) and frequency selective fading channels were addressed in [7]. In [21], a moment matching method was employed to obtain accurate closed-form approximations for AWGN and Rayleigh fading channels. Multi-antenna LoRa systems were addressed in [11,24]. The performance of relay-based LoRa networks was addressed in [25]. A first attempt to provide exact BER expressions for Rayleigh, Rice and Nakagami-*m* fading is available in [22]. Nevertheless, as was pointed out in [21,23], the proposed methodology for channels other than Rayleigh may suffer from numerical stability issues, due to the computation of large values of binomial coefficients. To this end, ref [23] leveraged the properties of the Marcum Q-function to provide accurate approximations for the BER of LoRa systems over Rice and Nakagami-*m* fading channels.

However, several results obtained using this method, i.e., for Nakagami-*m* fading, require the computation of hypergeometric functions with two arguments [23], which in turn are not available as built-in functions in standard mathematical software packages such as Matlab or Mathematica [26]. Moreover, for the numerical evaluation of the underlying mathematical expressions, the computation of an approximation threshold parameter is required. Nevertheless, the exact computation of this threshold is rather complicated and therefore, a heuristic method for its computation was proposed by the authors in the same work. The above facts motivate simpler, yet accurate expressions for the evaluation of the BER of LoRa systems in the presence of noise and fading. On the other hand, analytical results for the error performance of LoRa systems in the presence of fading channels other than Rayleigh, Rice and Nakagami-*m*, are—to the best of our knowledge—not available in the open technical literature. Indeed, as it was pointed out in [27], the above mentioned classical fading models do not always fit well measured data, especially at the tail portion. This motivates research on performance evaluation over generalized fading models that include the classical ones as special cases.

Motivated by the above facts, in this study we present new analytical expressions for the average bit error rate evaluation of LoRa systems in the presence of fading. More specifically, the novel research contributions of this work can be summarized as follows.

1. Under the assumption of Nakagami-*m* and Rice fading channels, we present approximate analytical expressions for the SER performance of LoRa systems. These expressions yield accurate results in the entire signal to noise ratio (SNR) region that are practically indistinguishable from the exact solution;
2. For the special case of Nakagami-*m* fading, using a moment matching method, a simple yet tight approximation to the SER is obtained in closed form;
3. For all fading scenarios, exact analytical SER expressions in terms of a single integral are presented;
4. A novel, accurate analytical expression for the SER of LoRa systems operating in the presence of Hoyt fading is presented. To this end, a new integral involving exponentials, modified Bessel functions and the Marcum-Q function, whose second argument is a linear function of the integration variable, is evaluated;
5. An exact single integral expression for the SER of LoRa systems operating over η-μ fading channel is presented, assuming a propagation environment consisting of a finite number of multi-path clusters;
6. An exact single integral expression for the SER of LoRa systems operating over generalized fading channels is presented, by approximating the PDF of the SNR with a mixture gamma distribution. As a test case, SER results of LoRa systems operating in the presence of κ-μ fading channels are presented.

In order to validate the correctness of the proposed mathematical analysis, all analytical results are substantiated by means of Monte-Carlo simulations. Note that the proposed analytical framework provides accurate results in the entire SNR range, thus circumventing the need for evaluating system performance via time consuming Monte-Carlo simulations (It is a common practice to use Monte-Carlo simulations in order to verify the correctness of analytical results. Please note that although Monte-Carlo simulations may also be used to obtain performance evaluation results, they suffer from two significant disadvantages, as compared to analytical results. First, one has to specify the system model using software defined components, i.e., to simulate channel, noise, modulation, demodulation and detection. Although this process provides further insights on the system structure, it is computationally very intensive, time consuming and requires large amounts of memory to achieve a given accuracy. Specifically, as a rule of thumb, in order to obtain exact BER results of the order of $10^{-6}$, random samples of two orders of magnitude larger, namely $10^{8}$, are required. Such large vectors are difficult to be handled by software tools such as Matlab. On the other hand, analytical results in the form of equations yield accurate results within a large range of system level parameters). The remainder of this work is structured as follows. Section 2 presents an overview of the LoRa modulation and its BER performance. Section 3 presents the main results of this work. Numerical results are presented in Section 4 whereas Section 5 concludes the work. *Notations:* A list of mathematical notations used in this work is available in Table A1.

## 2. Overview of the LoRa Modulation

In this section, an overview of the LoRa modulation and the corresponding bit error probability are presented. LoRa systems employ the shift chirp modulation scheme, also known as spread spectrum modulation. The number of samples within the duration of a symbol, $T_{s}$, is determined by the spreading factor (SF). It holds that $T_{s}=2^{\mathrm{SF}}/B$, where *B* is the signal bandwidth. In typical applications, SF∈{6,7,…,12}. Note that the coverage of LoRa is determined by SF. Specifically, increasing SF results in wider coverage but also in a reduction in the data rate.

The modulation encoder maps a group of SF bits to a symbol, $s_{k}$, k∈{0,1,…L} where $L=2^{\mathrm{SF}}-1$. The transmitted waveform can be expressed as [21]

(1) $$\begin{array}{cc} s_{k}\left(nT\right) & =h\sqrt{E_{s}}\omega_{k}\left(nT_{s}\right) \end{array}$$

(2) $$=h\sqrt{\frac{E_{s}}{N}}\exp\left\{j2\pi \frac{n}{N}\left[\left(k+n\right)\;\mathrm{mod}\;N\right]\right\}$$

where $N=2^{\mathrm{SF}}$, $T_{s}=1/B$ is the sampling period, n∈{0,1,…L} is the sample index at time $nT_{s}$, $E_{s}$ is the signal energy, *h* is the fading channel coefficient and $\omega_{k}\left(nT_{s}\right)$ are orthonormal basis functions.

**Figure 1:**
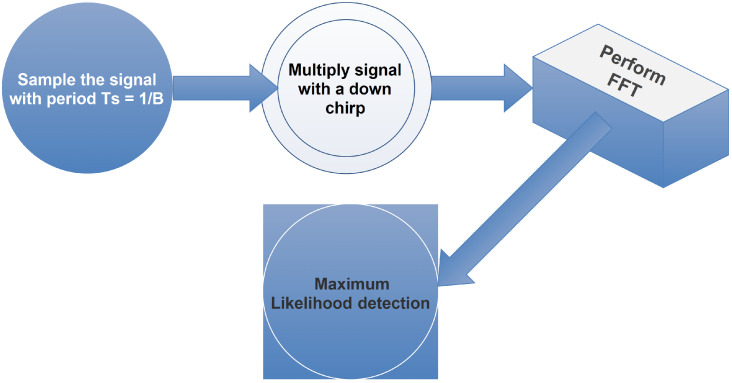
A simplified overview of the LoRa non-coherent demodulator.

Figure 1 depicts the main functional blocks of a LoRa non-coherent demodulator. Specifically:

- The input signal is sampled at a period of $T_{s}=1/B$;
- The resulting signal is then multiplied with a down chirp signal;
- A Fast Fourier Transform (FFT) is performed at the output of the previous block to retrieve the symbol value;
- The information signal is estimated using maximum likelihood detection.

Using the orthogonal properties of $s_{k}\left(nT\right)$, the correlator output at the demodulator is given as [21]

(3) $$\begin{array}{c} \sum_{n=0}^{L}r_{k}\left(nT\right)\omega_{i}^{*}\left(nT\right)=\left\{\begin{array}{cc} h\sqrt{E_{s}}+\phi_{i} & \mathrm{if}\;k=i\\ \phi_{i} & \mathrm{if}\;k\neq i \end{array}\right. \end{array}$$

where $r_{k}\left(\cdot \right)$ is the received signal and $\phi_{i}$ is the complex Gaussian noise. The decision rule for the detected index symbol can be expressed as [21]

(4) $$\begin{array}{c} \hat{k}=\left\{i|\arg_{i}\max\left(|\delta_{k,i}h\sqrt{E_{s}}+\phi_{i}|\right)\right\}. \end{array}$$

The conditional symbol error probability given the squared channel coefficient *h* is given as [21,22,23]

(5) $$\begin{array}{c} P\left(e|h\right)=\mathrm{Pr}\{\rho^{2}>|h\sqrt{E_{s}}+\phi_{i}{|}^{2}\} \end{array}$$

where $\rho^{2}={\left.\max\{|\phi_{i}|\}\right|}_{i\neq k}$ is the maximum of *L* independent and identically distributed (i.i.d) exponential random variables with CDF given by

(6) $$\begin{array}{c} F_{\rho^{2}}\left(x\right)={\left[1-\exp\left(-x/2\right)\right]}^{L}. \end{array}$$

Moreover, the RV $R≜|h\sqrt{E_{s}}+\phi_{i}{|}^{2}$ conditioned to $h^{2}$ follows a non-central chi-square distribution with PDF given by [21,22,23]

(7) $$\begin{array}{c} f_{R|h^{2}}\left(x\right)=\frac{1}{2}\exp\left(-\frac{x+2Nh^{2}\gamma }{2}\right)I_{0}\left(\sqrt{2Nh^{2}\gamma x}\right), \end{array}$$

where $\gamma =1/E\langle |\phi_{i}{|}^{2}\rangle$ is the SNR. The CDF of *R* conditioned to $h^{2}$ can be expressed in terms of the Marcum Q-function as

(8) $$\begin{array}{c} F_{R|h^{2}}\left(x\right)=1-Q_{1}\left(\sqrt{2Nh^{2}\gamma },\sqrt{x}\right). \end{array}$$

Finally, using (5)–(7), the average symbol error probability is given in terms of the following two-fold integral [21,22,23]

(9) $$\begin{array}{c} P_{s}=\frac{1}{2}\int_{0}^{\infty }\int_{0}^{\infty }\left\{1-{\left[1-\exp\left(-x/2\right)\right]}^{L}\right\}\\ \times \exp\left(-\frac{x+2Ny\gamma }{2}\right)I_{0}\left(\sqrt{2N\gamma xy}\right)f_{h^{2}}\left(y\right)dxdy. \end{array}$$

The resulting bit error probability can be expressed as [21,23]

(10) $$\begin{array}{c} P_{b}=\frac{2^{\mathrm{SF}-1}}{2^{\mathrm{SF}}-1}P_{s}. \end{array}$$

## 3. Main Results

In this section, exact analytical expressions for $P_{s}$ in terms of a single integral as well as accurate approximations will be obtained for Nakagami-*m*, Ricean and Hoyt fading channels.

### 3.1. Symbol Error Probability for Nakagami-*m* Fading Channels

Under Nakagami-*m* fading, the RV $h^{2}$ follows a gamma distribution with PDF given as [28]

(11) $$\begin{array}{c} f_{h^{2}}\left(y\right)=\frac{m^{m}}{\Gamma (m)}y^{m-1}\exp\left(-my\right) \end{array}$$

where m>0 is the fading parameter. For m=1, i.e., for Rayleigh fading, (11) reduces to the exponential distribution. An exact analytical expression for $P_{s}$ is given in the following proposition.

**Proposition** **1.** *The exact symbol error probability of LoRa systems under Nakagami-m fading in terms of a single integral is given as*

(12) $$\begin{array}{cc} P_{s}^{\mathrm{Nak}} & =\frac{1}{2}{\left(\frac{m}{N\gamma +m}\right)}^{m}\int_{0}^{\infty }e^{-x/2}\left[1-{\left(1-e^{-x/2}\right)}^{L}\right]\\ & \times L_{-m}\left[\frac{N\gamma x}{2(N\gamma +m)}\right]dx \end{array}$$

**Proof.**  By substituting (11) into (9) and changing the order of integration, a valid operation according to the Fubini theorem because the resulting integrals are convergent, one obtains

(13) $$\begin{array}{c} P_{s}=\frac{m^{m}}{2\Gamma (m)}\int_{0}^{\infty }e^{-x/2}\left[1-{\left(1-e^{-x/2}\right)}^{L}\right]\\ \times \left[\int_{0}^{\infty }y^{m-1}e^{-(N\gamma +m)y}I_{0}\left(\sqrt{2Nxy}\right)dy\right]dx. \end{array}$$

By employing [29] (Equation (3.15.1/2)) and [30] (Equation (8.972/1)), (12) is readily obtained, thus completing the proof.    □

Note that (12) yields the exact value of $P_{s}$ for arbitrary values of *m*. In addition, it converges rapidly due to its exponentially decaying kernel and can be evaluated numerically in an efficient manner using built-in routines available in popular mathematical software packages such as Matlab or Mathematica. In what follows, we derive accurate approximations for $P_{s}$, assuming both arbitrary and integer values of the fading parameter *m*. The following result holds.

**Proposition** **2.** *For arbitrary values of m, an accurate approximation for the $P_{s}$ of LoRa systems in the presence of Nakgami-m fading is given as*

(14) PsNak≈mNγ+mmexp(−x˜N/2)×∑n=1∞x˜Nn2nΓ(n+1)F11m;n+1;Nγx˜N2(Nγ+m)

*where as for integer values of m*

(15) $$\begin{array}{cc} P_{s}^{\mathrm{Nak}} & \approx 1-\frac{N\gamma }{N\gamma +m}\exp\left[-\frac{m{\tilde{x}}_{N}}{2(N\gamma +m)}\right]\\ & \times \sum_{n=0}^{m-1}\epsilon_{n}{\left(\frac{m}{N\gamma +m}\right)}^{n}L_{n}\left[-\frac{N\gamma {\tilde{x}}_{N}}{2(N\gamma +m)}\right] \end{array}$$

*where*

(16) $$\begin{array}{c} \epsilon_{n}=\left\{\begin{array}{cc} 1 & \mathrm{if}\;n<m-1\\ 1+\frac{m}{N\gamma } & \mathrm{if}\;n=m-1 \end{array}\right. \end{array}$$

*and*

(17) $$\begin{array}{c} {\tilde{x}}_{N}=2\sum_{n=1}^{N-1}n^{-1}. \end{array}$$

**Proof.**  Our starting point to the proof is (5) via which a generic expression for $P_{s}$ can be obtained. Observe that for large values of SF, i.e., for SF≥6, the RV $\rho^{2}$ can be replaced with its mean with sufficient accuracy. Note that this observation has also been reported in [21]. Consequently, using (5) and (8), $P_{s}$ can be approximated as

(18) $$P_{s}\approx 1-E_{h^{2}}\langle Q_{1}\left(\sqrt{2N\gamma h^{2}},\sqrt{{\tilde{x}}_{N}}\right)\rangle ,$$

where ${\tilde{x}}_{N}$ is the expectation of the RV $\rho^{2}$, which, by employing the memoryless property of the exponential distribution [21], can be deduced as (17). Using (11), the expectation in (18) can be further written as

(19) $$\begin{array}{c} P_{s}\approx 1-\frac{m^{m}}{\Gamma (m)}\int_{0}^{\infty }y^{m-1}e^{-my}Q_{1}\left(\sqrt{2N\gamma y},\sqrt{{\tilde{x}}_{N}}\right)dy \end{array}$$

Using [31] (Equation (10)) and [31] (Equation (11)) (Note that [31] (Equation (11)) has a typo, i.e., *N* should be replaced with Γ(N)), (14) and (15) can be deduced for real and integer values of *m*, respectively, thus completing the proof.    □

Next, using a moment matching method, a simpler closed-form expression for $P_{s}$ will be derived, which holds for arbitrary values of *m*. Specifically, we propose approximating the statistics of the RV *R* with those of a gamma distribution with scale parameter *a* and shape parameter *b*, using a moment matching method. The following result holds.

**Proposition** **3.** *A closed-form approximation for the $P_{s}$ of LoRa systems under Nakagami-m fading can be obtained as*

(20) $$\begin{array}{c} P_{s}^{\mathrm{Nak}}\approx 1-\frac{\Gamma (a,b{\tilde{x}}_{N})}{\Gamma (a)}. \end{array}$$

*where ${\tilde{x}}_{N}$ is given by* (17),

(21a) $$a={\tilde{\mu }}_{1}^{2}/\left({\tilde{\mu }}_{2}-{\tilde{\mu }}_{1}^{2}\right),\;b={\tilde{\mu }}_{1}/\left({\tilde{\mu }}_{2}-{\tilde{\mu }}_{1}^{2}\right),$$

(21b) $${\tilde{\mu }}_{1}=2\left(1+N\gamma \right),$$

(21c) $${\tilde{\mu }}_{2}=8\left(1+2N\gamma \right)+4\gamma^{2}\left(1+m\right)N^{2}/m.$$

**Proof.**  Observe that $R^{2}$ follows a squared gamma-shadowed Rice distribution and thus, using [32] (Equation (5)), its *n*-moment is readily obtained as

(22) $$\begin{array}{cc} {\tilde{\mu }}_{n} & =2^{n}{\left(\frac{m}{N\gamma +m}\right)}^{m}\Gamma \left(n+1\right)\\ & \times_{2}F_{1}\left(m,n+1;1;\frac{N\gamma }{N\gamma +m}\right). \end{array}$$

Using [33] (Equation (7.3.1/129)), ${\tilde{\mu }}_{1}$ and ${\tilde{\mu }}_{2}$ can be further simplified as (21). Finally, $P_{s}$ can be deduced as the CDF of a gamma distribution with parameters *a* and *b* that can be obtained in closed form using a moment matching method [34] as (20) and (21), thus completing the proof.    □

Using Proposition 2, a closed-form approximation for the $P_{s}$ under Rayleigh fading will be obtained. Specifically, the following result holds.

**Corollary.**  *Under Rayleigh fading, a closed-form approximation for $P_{s}$ can be deduced as*

(23) $$\begin{array}{c} P_{s}^{\mathrm{Ray}}\approx 1-\exp\left[-\frac{{\tilde{x}}_{N}}{2(1+N\gamma )}\right] \end{array}$$

**Proof.**  The proof can be readily obtained by setting m=1 to (15).    □

### 3.2. Symbol Error Probability for Rice Fading Channels

Under Rice fading, the RV $h^{2}$ follows a non-central chi-square distribution with PDF given as [28]

(24) $$\begin{array}{c} f_{h^{2}}\left(y\right)=\frac{1+K}{\exp(K)}\exp\left[-(1+K)y\right]I_{0}\left[2\sqrt{K(1+K)y}\right], \end{array}$$

where *K* is the Rice factor. For K=0, (24) reduces to the exponential distribution, i.e., Rayleigh fading.

An exact analytical expression for $P_{s}$ is given in the following proposition.

**Proposition** **4.** *The exact symbol error probability of LoRa systems under Rice fading in terms of a single integral is given as*

(25) $$\begin{array}{cc} P_{s}^{\mathrm{Rice}} & =\frac{\left(1+K)\exp(-K\right)}{2(1+K+N\gamma )}\int_{0}^{\infty }e^{-x/2}\left[1-{\left(1-e^{-x/2}\right)}^{L}\right]\\ & \times e^{\frac{2K+2K^{2}+\gamma Nx}{2+2K+2\gamma N}}I_{0}\left[\frac{\sqrt{2NK(1+K)\gamma x}}{1+K+N\gamma }\right]dx \end{array}$$

**Proof.**  The proof can be concluded by following a similar line of arguments as in the proof of Proposition 1. Specifically, by substituting (24) into (9) and changing the order of integration, $P_{s}$ can be expressed as

(26) $$\begin{array}{c} P_{s}=\frac{1+K}{2\exp(K)}\int_{0}^{\infty }e^{-x/2}\left[1-{\left(1-e^{-x/2}\right)}^{L}\right]\\ \times \left[\int_{0}^{\infty }e^{-(N\gamma +K+1)y}I_{0}\left[2\sqrt{K(1+K)y}\right]\right.\\ \times \left.I_{0}\left(\sqrt{2Nxy}\right)dy\right]dx. \end{array}$$

The inner integral, i.e., with respect to *y*, can be evaluated in closed form by employing [29] (Equation (3.15.17/1)), yielding (25), thus completing the proof.    □

Again, (25) converges rapidly due to its exponentially decaying kernel and can be evaluated numerically in an efficient manner. In what follows, an accurate approximation for $P_{s}$ will be derived. The following result holds.

**Proposition** **5.** *Under Rice fading, an accurate closed-form approximation for $P_{s}$ can be deduced as*

(27) $$\begin{array}{c} P_{s}^{\mathrm{Rice}}\approx 1-Q_{1}\left[\sqrt{\frac{2N\gamma K}{1+K+N\gamma }},\sqrt{\frac{{\tilde{x}}_{N}\left(1+K\right)}{1+K+N\gamma }}\right] \end{array}$$

**Proof.**  Using (18) and (24), $P_{s}$ can be approximated as

(28) $$\begin{array}{cc} P_{s}\approx & 1-\frac{1+K}{\exp(K)}\int_{0}^{\infty }e^{-(1+K)y}I_{0}\left[2\sqrt{K(1+K)y}\right]\\ & \times Q_{1}\left(\sqrt{2N\gamma y},\sqrt{{\tilde{x}}_{N}}\right)dy \end{array}$$

By employing [35] (Equation (15)), the resulting integral can be evaluated in closed-form yielding (27), thus completing the proof.    □

### 3.3. Symbol Error Probability for Hoyt Channels

Under Hoyt (Nakagami-*q*) fading, the RV $h^{2}$ follows a non-central chi-square distribution with PDF given as [28]

(29) $$\begin{array}{cc} f_{h^{2}}\left(y\right) & =\sqrt{\frac{1}{2}+\frac{1}{4\eta }+\frac{\eta }{4}}\exp\left[-\left(\frac{1}{2}+\frac{1}{4\eta }+\frac{\eta }{4}\right)y\right]\\ & \times I_{0}\left[\left(\frac{1}{4\eta }-\frac{\eta }{4}\right)y\right], \end{array}$$

where $\eta =q^{2}$, with 0<q≤1 being a parameter related to the fade intensity. For q=1, (29) reduces to the exponential distribution (Rayleigh fading).

An exact expression for $P_{s}$ can be deduced using the following proposition.

**Proposition** **6.** *Under Hoyt fading, $P_{s}$ can be expressed in terms of a single integral as*

(30) $$\begin{array}{cc} P_{s}^{\mathrm{Hoyt}} & =\frac{\eta +1}{2\sqrt{A}}\int_{0}^{\infty }e^{-x/2}\left[1-{\left(1-e^{-x/2}\right)}^{L}\right]\\ & \times e^{\frac{N\gamma x[{\left(1+\eta \right)}^{2}+4N\eta \gamma ]}{2A}}I_{0}\left[\frac{N(\eta^{2}-1)\gamma x}{2A}\right]dx \end{array}$$

*where*

(31) $$\begin{array}{c} A=1+2N\gamma +\eta^{2}\left(1+2N\gamma \right)+2\eta \left(1+2N\gamma +2N^{2}\gamma^{2}\right) \end{array}$$

**Proof.**  The proof can be concluded by following a similar line of arguments as in the proof of Proposition 1. Specifically, by substituting (29) into (9) and changing the order of integration, $P_{s}$ can be expressed as

(32) $$\begin{array}{c} P_{s}=\frac{1}{2}\sqrt{\frac{1}{2}+\frac{1}{4\eta }+\frac{\eta }{4}}\int_{0}^{\infty }e^{-x/2}\left[1-{\left(1-e^{-x/2}\right)}^{L}\right]\\ \times \left\{\int_{0}^{\infty }e^{-\left(N\gamma +\frac{1}{2}+\frac{1}{4\eta }+\frac{\eta }{4}\right)y}I_{0}\left[\left(\frac{1}{4\eta }-\frac{\eta }{4}\right)y\right]\right.\\ \times \left.I_{0}\left(\sqrt{2Nxy}\right)dy\right\}dx. \end{array}$$

The inner integral, i.e., with respect to *y*, can be evaluated in closed form by employing [29] (Equation (3.15.17/15)), yielding (30), thus completing the proof.    □

An accurate approximation for $P_{s}$ can be obtained using the following proposition. The following result holds.

**Proposition** **7.** *Under Hoyt fading, an accurate approximation for $P_{s}$ can be deduced as*

(33) $$\begin{array}{c} P_{s}^{\mathrm{Hoyt}}\approx 1-\frac{1}{\gamma }\sqrt{\frac{1}{4\eta }+\frac{\eta }{4}+\frac{1}{2}}\\ \times I\left[\sqrt{2N},\sqrt{{\tilde{x}}_{N}},\frac{1}{\gamma }\left(\frac{1}{4\eta }-\frac{\eta }{4}\right),\frac{1}{\gamma }\left(\frac{1}{4\eta }+\frac{\eta }{4}+\frac{1}{2}\right)\right] \end{array}$$

*where*

(34) $$\begin{array}{cc} I(a,b,c,p) & =\frac{a^{2}}{2p+a^{2}}\exp\left[-\frac{b^{2}p}{2p+a^{2}}\right]\\ & \times \sum_{k=0}^{\infty }\sum_{n=0}^{2k}\frac{\Gamma \left(2k+1\right)c^{2k}}{p^{2k+1}{\left(k!\right)}^{2}4^{k}}\\ & \times \zeta_{n,k}{\left(\frac{2p}{2p+a^{2}}\right)}^{n}L_{n}\left[-\frac{b^{2}a^{2}}{4p+2a^{2}}\right] \end{array}$$

*and*

(35) $$\begin{array}{c} \zeta_{n,k}=\left\{\begin{array}{cc} 1 & \mathrm{if}\;n<2k\\ 1+\frac{2p}{a^{2}} & \mathrm{if}\;n=2k \end{array}\right. \end{array}$$

**Proof.**  Using (18) and (29), $P_{s}$ can be approximated as

(36) $$\begin{array}{cc} P_{s}\approx & 1-\sqrt{\frac{1}{2}+\frac{1}{4\eta }+\frac{\eta }{4}}\int_{0}^{\infty }e^{-\left(\frac{1}{2}+\frac{1}{4\eta }+\frac{\eta }{4}\right)y}\\ & \times I_{0}\left[\left(\frac{1}{4\eta }-\frac{\eta }{4}\right)y\right]Q_{1}\left(\sqrt{2N\gamma y},\sqrt{{\tilde{x}}_{N}}\right)dy \end{array}$$

which can be written as (33) with

(37) $$\begin{array}{c} I\left(a,b,c,p\right)=\int_{0}^{\infty }\exp\left(-px\right)I_{0}\left(cx\right)Q_{1}\left(a\sqrt{x},b\right)dx \end{array}$$

To the best of our knowledge, however, this integral is not available in related works such as [31,35,36]. Nevertheless, as shown in the Appendix A, I(a,b,c,p) can be evaluated as (34), thus completing the proof.    □

### 3.4. Symbol Error Probability for Physical η-μ Fading Channels

Under η-μ fading, the PDF of $h^{2}$ is given by [27]

(38) $$f_{h^{2}}\left(y\right)=\frac{2\sqrt{\pi }\mu^{\mu +0.5}\theta^{\mu }y^{\mu -0.5}}{\Gamma \left(\mu \right)H^{\mu -0.5}}\exp\left(-2\mu \theta y\right)I_{\mu -0.5}\left(2\mu_{\ell }H\gamma \right)$$

where μ is related to the fading severity. The η-μ fading is quite general as it can accurately model small-scale variations of the fading signal under non line-of-sight (NLOS) conditions and includes as special cases both the Nakagami-*m* and the Hoyt fading models. The PDF of $h^{2}$ may be expressed in two formats, namely Format 1, where $\theta =(2+\eta^{-1}+\eta )/4$ and $H=(\eta^{-1}-\eta )/4$ with 0<η<∞ and Format 2, where $\theta =1/(1-\eta^{2})$ and $H=\eta /(1-\eta^{2})$ with −1<η<1. As pointed out in [27], Format 1 can be converted into Format 2 by employing a bilinear transformation. Thus and without loss of generality, Format 1 will be assumed next. Moreover, the special case of integer μ, termed *physical* η-μ model, assumes a finite number of multipath clusters and has been adopted in several works, e.g., see [27,37,38,39]

Using [30] (Equation (8.467)), the modified Bessel function $I_{\pm (n+1/2)}\left(z\right)$, with n>0 being an integer, can be expressed as a finite sum, namely

(39) $$I_{\pm (n+1/2)}\left(z\right)=\frac{1}{\sqrt{\pi }}\sum_{k=0}^{n}\frac{(n+k)!}{n!(n-k)!}\left[\frac{{\left(-1\right)}^{k}e^{z}\mp {\left(-1\right)}^{n}e^{-z}}{{\left(2z\right)}^{k+0.5}}\right].$$

Substituting (39) into (38) and employing [30] (Equation (8.353/7)), $f_{h^{2}}\left(y\right)$ can be expressed as

(40) $$f_{h^{2}}\left(y\right)=\frac{\mu \theta }{H\Gamma (\mu )}\sum_{k=0}^{\mu -1}a_{k}y^{\mu -k-1}\left[{\left(-1\right)}^{k}e^{-Ay}+{\left(-1\right)}^{\mu }e^{-By}\right]$$

where

(41a) $$a_{k}=\frac{{\left(-1\right)}^{k}\left(\mu +k-1\right)!{\left(4\mu H\right)}^{-k}}{k!(\mu -k-1)!}$$

(41b) A=2μ(θ−H),B=2μ(θ+H).

An exact expression for $P_{s}$ can be obtained using the following proposition.

(42) $$\begin{array}{c} P_{s}^{\eta -\mu }=\frac{0.5}{\Gamma (\mu )}{\left(\frac{\mu \theta }{H\gamma }\right)}^{\mu }\left\{\sum_{k=0}^{\mu -1}a_{k}\gamma^{k}{\left(-1\right)}^{k}\Gamma \left(\mu -k\right)2^{\mu -k}\left[{\left(-1\right)}^{k}{\left(\lambda +\frac{2A}{\gamma }\right)}^{k-\mu }\right.\right.\\ \times \int_{0}^{\infty }e^{-x/2}\left[1-{\left(1-e^{-x/2}\right)}^{L}\right]L_{k-\mu }\left[\frac{\lambda \gamma x}{2(\lambda \gamma +2A)}\right]dx\\ +{\left(-1\right)}^{\mu }{\left(\lambda +\frac{2B}{\gamma }\right)}^{k-\mu }\int_{0}^{\infty }e^{-x/2}\left[1-{\left(1-e^{-x/2}\right)}^{L}\right]L_{k-\mu }\left[\frac{\lambda \gamma x}{2(\lambda \gamma +2B)}\right]dx. \end{array}$$

**Proposition** **8.** *The exact symbol error probability of LoRa systems operating under* physical *η-μ fading channels can be expressed in terms of a single integral as* (42).

**Proof.**  The proof can be readily deduced by following the same steps as in the proof of Proposition 1.    □

### 3.5. Symbol Error Probability for Generalized Fading Channels Using a Mixture Gamma Distribution

In what follows, we present analytical results for the SER of LoRa systems assuming generalized fading channels for which the PDF of the SNR can be expressed as a mixture gamma distribution. As was shown in [40], the proposed approach is valid for a plethora of fading distributions, including the κ-μ, the η-μ and composite fading/shadowing channels such as the generalized-K and the Suzuki ones. The PDF of $h^{2}$ can be expressed as [40] (Equation (1))

(43) $$\begin{array}{c} f_{h^{2}}\left(y\right)=\sum_{i=1}^{N_{\mathrm{terms}}}a_{i}y^{\beta_{i}-1}e^{-\zeta_{i}y}, \end{array}$$

where $N_{\mathrm{terms}}$ is the number of terms required for a given accuracy, and $a_{i}$, $\beta_{i}$ and $\zeta_{i}$ are the parameters of the ith Gamma component. The parameter $N_{\mathrm{terms}}$ can be selected so that the first *k* moments of the original and the approximate distributions are matched or the Kullback–Leibler distance of the original and the approximate distributions is minimized [40]. For such channels, an exact expression for $P_{s}$ can be obtained using the following proposition.

**Proposition** **9.** *The exact symbol error probability of LoRa systems operating under generalized fading channels can be expressed in terms of a single integral as*

(44) $$\begin{array}{cc} P_{s}^{\mathrm{gen}} & =\frac{1}{2}\sum_{i=1}^{N_{\mathrm{terms}}}a_{i}{\left(N\gamma +\zeta_{i}\right)}^{-\beta_{i}}\Gamma \left(\beta_{i}\right)\int_{0}^{\infty }e^{-x/2}\\ & \times \left[1-{\left(1-e^{-x/2}\right)}^{L}\right]L_{-\beta_{i}}\left[\frac{N\gamma x}{2(N\gamma +\zeta_{i})}\right]dx \end{array}$$

**Proof.**  The proof can be readily deduced by following the same steps as in the proof of Proposition 1.    □

## 4. Numerical Results

In this section, numerical results are presented to validate the proposed error rate analysis. Analytical results are compared with equivalent ones obtained using Monte-Carlo simulations. A number of random samples equal to $10^{5}$ is used to ensure statistical convergence. The simulation methodology is described in Algorithm  1. Unless otherwise specified, values of SF of 7 and 12 have been assumed.
**Algorithm 1** Monte-Carlo simulation methodology.**Require:** Number of samples ≥0
$E_{s}\leftarrow \mathrm{SNR}$errors←0Number of samples $\leftarrow 10^{5}$**while** Number of samples ≠0 **do** generate random channel coefficient *h* for a given fading distribution generate noise coefficient $\phi_{i}$ from a normal distribution generate $\rho^{2}$ as the maximum of exponential random variables **if** $\rho^{2}>{\left|h\sqrt{E_{s}}+\phi_{i}\right|}^{2}$ **then**  errors←errors+1**end if**Numberofsamples←Numberofsamples−1**end while**


**Figure 2:**
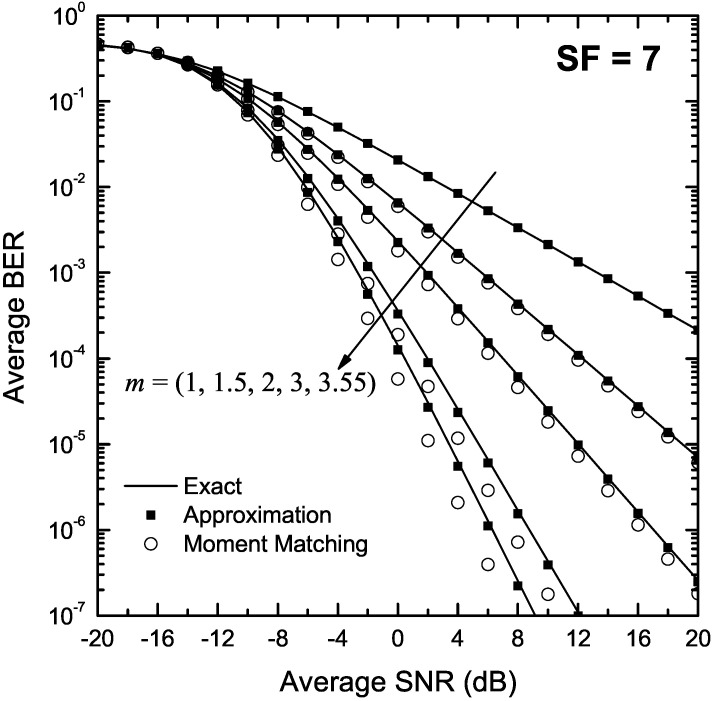
BER of LoRa systems operating in the presence of Nakagami-*m* fading as a function of the SNR, γ, for SF = 7 and various values of *m*.

**Figure 3:**
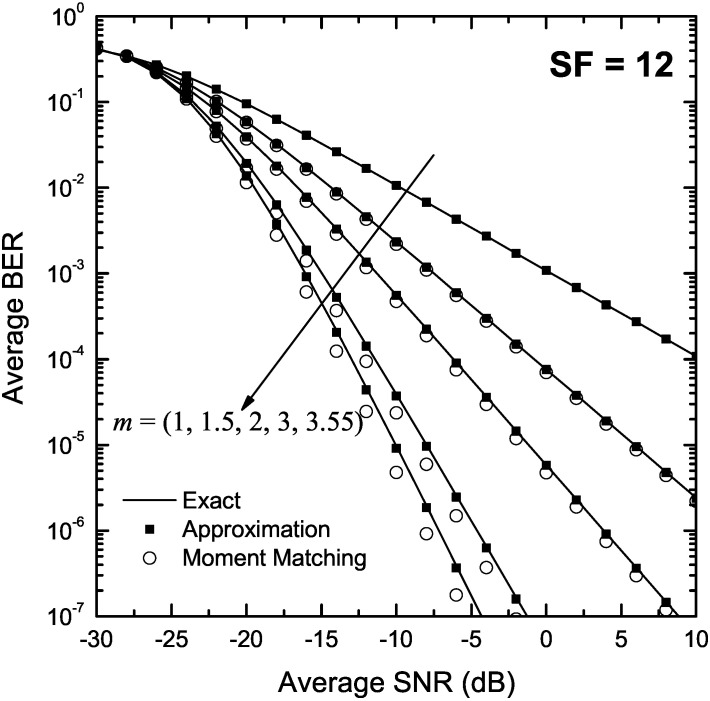
BER of LoRa systems operating in the presence of Nakagami-*m* fading as a function of the SNR, γ, for SF = 12 and various values of *m*.

Figure 2 and Figure 3 depict the BER of LoRa modulation in the presence of Nakagami-*m* fading as a function of γ for m∈{1,1.5,2,3,3.55}, and SF of 7 and 12, respectively. In both figures, approximate BER results for m>1 were obtained using the approximation presented in Proposition 2 as well as the moment matching method in Proposition 3. The exact BER values were obtained using the two-fold integral in (9), the single integral expression in Proposition 1 and Monte-Carlo simulation based on (5), using $10^{5}$ random samples. For m=1, i.e., Rayleigh fading, approximate results were obtained using (23). As it can be observed, the approximate formulas obtained using Proposition 2 match well the exact results in the entire SNR region. In addition, the moment matching method yields accurate results for low values of *m*; nevertheless, deviations from the exact results are observed for m>2 and high SNR values. Finally, for Rayleigh fading, (23) yields very accurate results that are practically indistinguishable from the exact ones.

**Figure 4:**
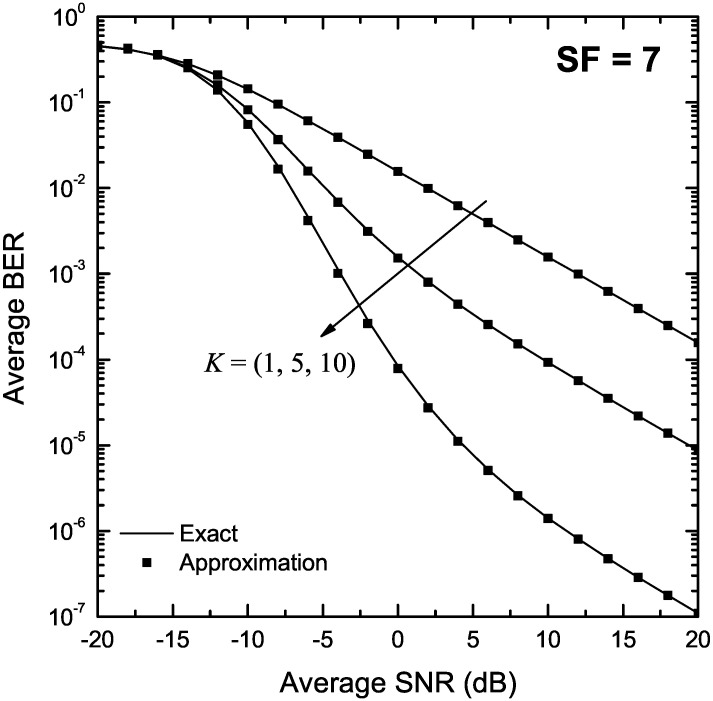
BER of LoRa systems operating in the presence of Rice fading as a function of the SNR, γ, for SF = 7 and various values of *K*.

**Figure 5:**
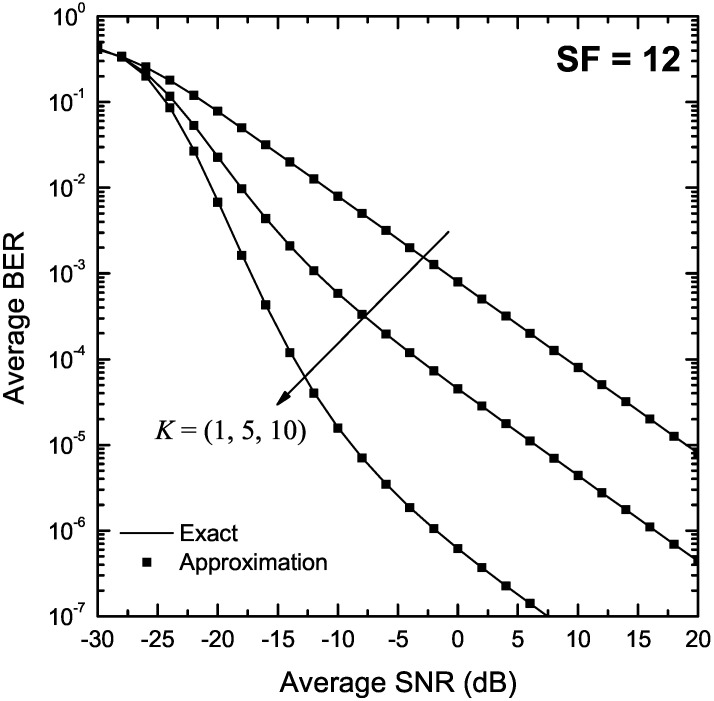
BER of LoRa systems operating in the presence of Rice fading as a function of the SNR, γ, for SF = 12 and various values of *K*.

Figure 4 and Figure 5 depict the BER of LoRa modulation in the presence of Rice fading as a function of γ for K∈{1,5,10}, and SF of 7 and 12, respectively. The exact BER values were obtained using both the two-fold integral in (9) as well as the single integral expression in Proposition 4. In both figures, approximate BER results have also been obtained using the approximation presented in Proposition 5. As it can be observed, the approximate formulas obtained using Proposition 2 match well the exact results in the entire SNR region for all values of *K*.

**Figure 6:**
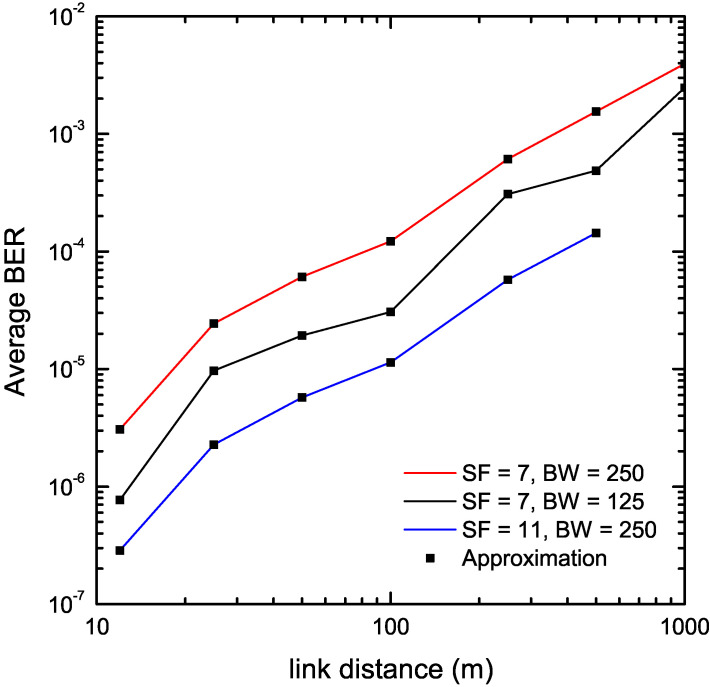
BER estimation of LoRa systems operating in the presence of Rice fading in an agricultural environment using a measurement campaign.

Next, we estimate the BER of LoRa systems in an agricultural environment, described in detail in [41]. In that work, an experimental test bed exploiting smartphone components was utilized in a measurement campaign, performed under realistic, in terms of agriculture, conditions. Specifically, part of the measurement campaign focused on measuring the Received Signal Strength Indicator (RSSI) for various distances between the LoRa radio modules participating in the experiments and for various transmit power level settings. Both LoRa radios had their transmit power adjusted to 10 dBm, the SF was set to either 7 or 11 and the bandwidth (BW) was 125 kHz or 250 kHz. It has also further been assumed that small scale fading is modeled by the Rice distribution with K=2.63 dB, a typical value encountered in rural environments [42]. The noise power equals −85 dBm. Figure 6 depicts the estimated BER of the considered propagation scenario as a function of the link distance. Again, an excellent match of exact and approximate results was observed for all test cases under consideration.

**Figure 7:**
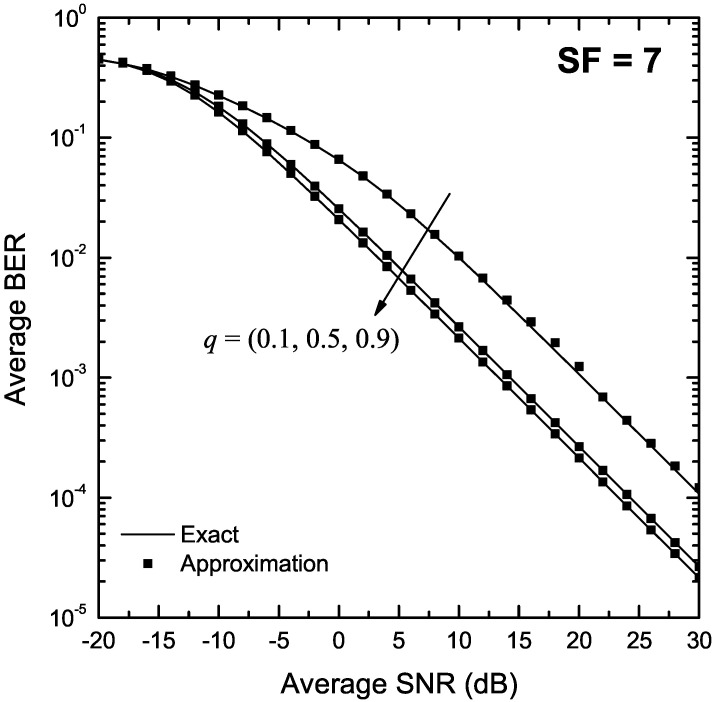
BER of LoRa systems operating in the presence of Hoyt fading as a function of the SNR, γ, for SF = 7 and various values of *q*.

**Figure 8:**
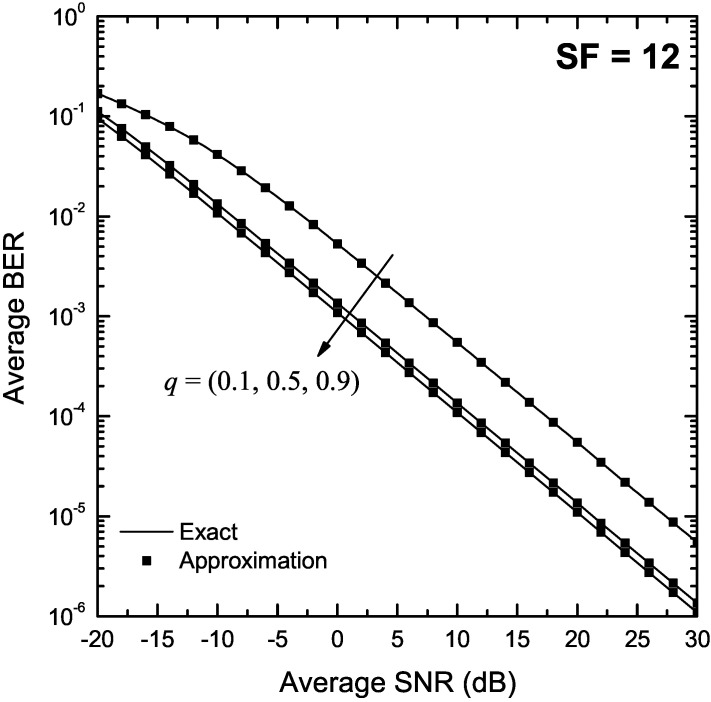
BER of LoRa systems operating in the presence of Hoyt fading as a function of the SNR, γ, for SF = 12 and various values of *q*.

Figure 7 and Figure 8 depict the SER of LoRa modulation in the presence of Hoyt fading as a function of γ for q∈{0.1,0.5,0.9}, and SF of 7 and 12, respectively. Again, the exact BER values were obtained using both the two-fold integral in (9) and the single integral expression in Proposition 6. The approximate BER results were obtained using Proposition 7. In order to derive the approximate BER results, the corresponding infinite series were truncated to N=220 and N=350 terms for q=0.1 and SF of 7 and 12, respectively whereas for q=0.5 and q=0.9, only 20 terms were sufficient to provide a good match with the analytical results for both considered values of SF. Again, the approximate formulas obtained using Proposition 7 match well with the exact results in the entire SNR region for all values of *q*.

**Figure 9:**
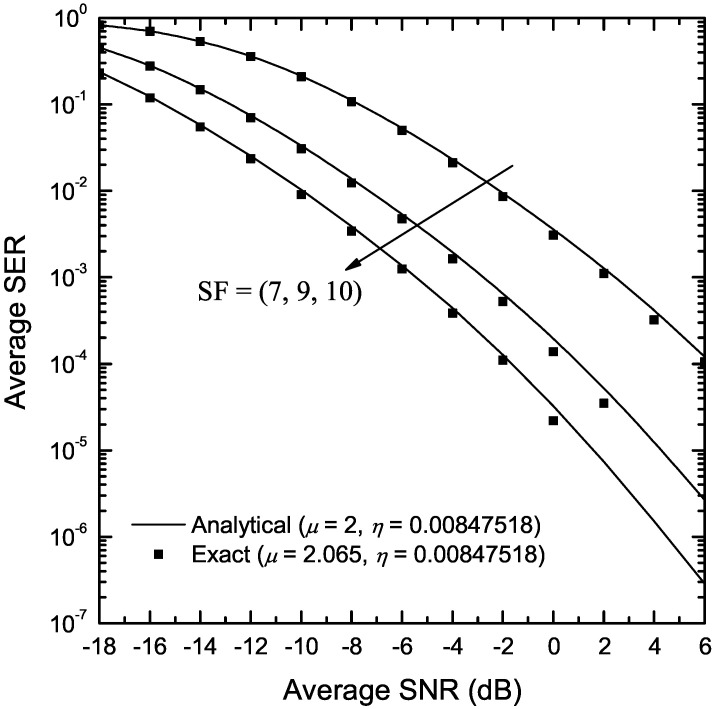
SER of LoRa systems operating in the presence of η-μ fading as a function of the SNR, γ, in an indoor environment, as reported in [27] and various values of SF.

Figure 9 depicts the SER of LoRa modulation in the presence of η-μ fading as a function of γ for SF∈{7,9,10}. The fading parameters are assumed to be μ=2.065 and η=0.00847518, obtained through a measurement campaign in an indoor environment, as reported in [27]. In order to apply the analytical results obtained in Proposition 8, a *physical* η-μ model with μ=2 was assumed. Exact results were obtained using Monte-Carlo simulation. As can be observed, analytical results closely approximate exact ones, for all considered values of SF, especially for low and medium values of γ, thus demonstrating the usefulness of the proposed analysis.

In the following, we consider LoRa systems operating in the presence of κ-μ fading as a function of γ. Note that the κ-μ distribution is a two-parameter fading model that well describes wireless propagation in the presence of a line-of-sight (LoS) component [27]. The PDF of $h^{2}$ is given by [27]

(45) $$\begin{array}{c} f_{h^{2}}\left(y\right)=\frac{\mu {\left(1+\kappa \right)}^{0.5(\mu +1)}y^{0.5(\mu_{X,k}-1)}}{\kappa^{0.5(\mu -1)}\exp\left(\mu \kappa \right)}\\ \times \exp\left[-\mu (1+\kappa )y\right]I_{\mu -1}\left[2\mu \sqrt{\kappa (1+\kappa )y}\right] \end{array}$$

where κ and μ account for the intensity of the LoS component and the Nakagami-*m* component, respectively. Note that the κ-μ distribution includes both the Nakagami-*m*(κ=0) and the Rice (m=1) distributions as special cases.

Using [40] (Equation (19)), the parameters of its mixture gamma approximation can be expressed as

(46a) $$a_{i}=\psi \left(\theta_{i},\beta_{i},\zeta_{i}\right),\;\beta_{i}=\mu +i-1,\;\zeta_{i}=\mu \left(1+\kappa \right)$$

(46b) $$\theta_{i}=\frac{\mu {\left(1+\kappa \right)}^{0.5(\mu +1)}}{\kappa^{0.5(\mu -1)}\exp\left(\mu \kappa \right)}\frac{\mu^{2i+\mu -3}{\left[\kappa \left(1+\kappa \right)\right]}^{0.5(2i+\mu -3)}}{\left(i-1)!\Gamma (\mu +i-1\right)}$$

(46c) $$\psi \left(\theta_{i},\beta_{i},\zeta_{i}\right)=\frac{\theta_{i}}{\sum_{j=1}^{N_{\mathrm{terms}}}\theta_{j}\Gamma \left(\beta_{j}\right)\zeta_{j}^{-\beta_{j}}}.$$

**Figure 10:**
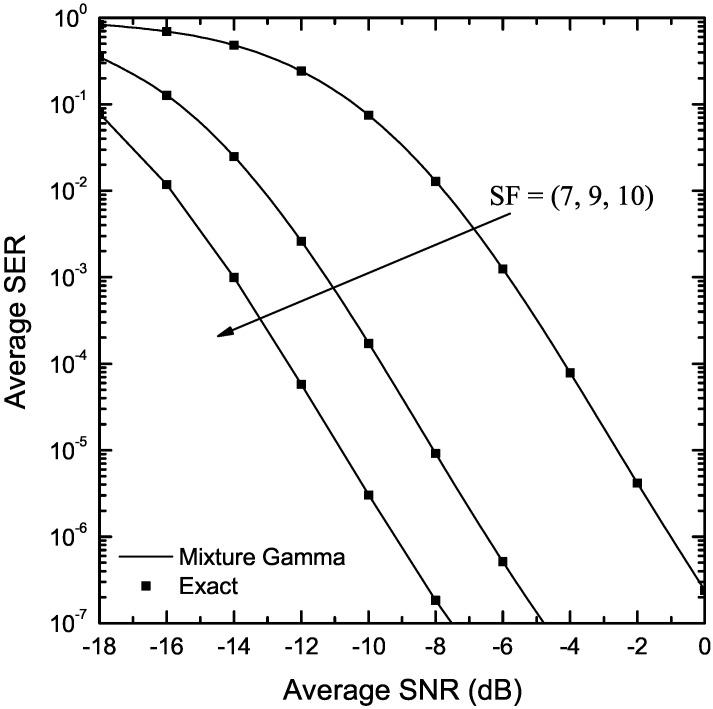
SER of LoRa systems operating in the presence of κ-μ fading as a function of the SNR, γ, for κ=10, μ=2.1 and various values of SF.

Figure 10 depicts the SER of LoRa systems over κ-μ fading, assuming μ=2.1, κ=10 and SF∈{7,9,10}. The κ-μ distribution was approximated with a mixture gamma distribution using 37 terms. As it is evident, the results obtained using the mixture gamma approximation match very well with the exact ones, obtained using (9) and (45), for all considered values of SF.

Finally, it is worth pointing out that our newly derived formulae for Rice and Nakagami-*m* fading were tested against the ones proposed in [23] and a close match was reported. Nevertheless, as also mentioned in the introduction section, the proposed analytical framework still provides accurate results with much lower complexity than those reported in [23].

## 5. Conclusions

In this work, we elaborated the LoRa system model to include an extensive performance analysis in the presence of various types of fading channels, using exact single integral expressions as well as accurate approximations. The results presented herein are valid for most of the well-known fading models available in the open technical literature. Moreover, they are computationally efficient and thus they may serve as a useful tool for system engineers for performance evaluation purposes.

## Figures and Tables

**Table 1:** Related works on theoretical performance of LoRa systems in the presence of fading and noise.

Authors	Title	Source	Findings
Vangelista, L.	Frequency shift chirp modulation: The LoRa modulation	[7]	Introduced the LoRa modulation system and provided initial results on its performance over AWGN channels by means of a single integral.
Elshabrawy, T.; Robert, J.	Closed-form approximation of LoRa modulation BER performance	[21]	Provided simple closed-form expressions of LoRa systems in the presence of AWGN and Rayleigh fading.
Dias, C.F.; Lima, E.R.D.; Fraidenraich, G.	Bit error rate closed-form expressions for LoRa systems under Nakagami and Rice fading channels.	[22]	Provided an exact closed-form expression for the BER of LoRa systems under Rayleigh fading as well as analytical expressions for the BER under Nakagami-*m* and Rice fading in terms of a finite sum.
Courjault, J.; Vrigenau, B.; Berder, O.; Bhatnagar, M.	A Computable Form for LoRa Performance Estimation: Application to Ricean and Nakagami Fading.	[23]	Authors elaborate on the properties of the generalized Marcum Q-function to provide accurate expressions for the BER of LoRa systems in the presence of Rice and Nakagami-*m* fading.
Hoeller, A.; et al.	Analysis and Performance Optimization of LoRa Networks With Time and Antenna Diversity	[11]	Authors addressed the performance of LoRa systems operating in the presence of Rayleigh fading, enhanced with antenna and time diversity techniques. The optimization of the performance of such systems has further been addressed.
Ma, H.; Cai, G.; Fang, Y.; Chen, P.; Han, G.	Design and Performance Analysis of a New STBC-MIMO LoRa System	[24]	Authors have proposed a new STBC MIMO LoRa system architecture. Its theoretical performance was analyzed in the presence of Rayleigh fading. A closed-form approximate BER expression of the proposed system under perfect and imperfect channel state information (CSI) was proposed.
Xu, W.; Cai, G.; Chen,	Performance analysis of a two-hop relaying LoRa system	[25]	Authors studied a two-hop opportunistic amplify-and-forward relaying LoRa system employing a best relay-selection protocol and operating over Nakagami-*m* fading.

## Data Availability

Data can be available upon mail request.

## Appendix A. Evaluation of I(a,b,c,p)

In order to obtain an analytical expression for I(a,b,c,p), we first employ an infinite series representation for the modified Bessel function, namely [30] (Equation (8.447/1))

(A1) $$\begin{array}{c} I_{0}\left(cx\right)=\sum_{n=0}^{\infty }\frac{c^{2k}x^{2k}}{2^{2k}{\left(k!\right)}^{2}} \end{array}$$

Substituting (A1) into (37) and exchanging the series and integral operators—a valid operation due to the uniform convergence of the resulting integrals—I(a,b,c,p) can be written as

(A2) $$\begin{array}{cc} I(a,b,c,p) & =\sum_{n=0}^{\infty }\frac{c^{2k}}{2^{2k}{\left(k!\right)}^{2}}\int_{0}^{\infty }x^{2k}\exp\left(-px\right)\\ & Q_{1}\left(a\sqrt{x},b\right)dx. \end{array}$$

Because 2k is always an integer, (A2) can be evaluated using [31] (Equation (11)), yielding (A2), thus completing the proof.

**Table A1:** Mathematical Notations.

$𝒥=\sqrt{-1}$	imaginary unit
$z^{*}$	conjugate of the complex number *z*
$\mathrm{Pr}\left\{\cdot \right\}$	probability operator
$E_{X}\langle \cdot \rangle$	expectation of the random variable (RV)
$f_{X}\left(\cdot \right)$	probability density function of the RV *X*
$F_{X}\left(\cdot \right)$	cumulative distribution function of the RV *X*
$\delta_{i,k}$	Kronecker delta function: $\delta_{i,k}=1$ for i=k and 0 otherwise
$I_{a}\left(\cdot \right)$	modified Bessel function of the first kind and order *a* [30] (Equation (8.431))
$\Gamma \left(\cdot \right)$	Gamma function [30] (Equation (8.310/1))
$\Gamma \left(\cdot ,\cdot \right)$	incomplete Gamma function [30] (Equation (8.350/2))
${}_{p}F_{q}\left(\cdot \right)$	generalized hypergeometric function [30] (Equation (9.14/1))
$Q_{m}\left(\cdot \right)$	generalized Marcum-Q function [35]: $Q_{m}\left(a,b\right)=a^{1-m}\int_{b}^{\infty }x^{m}\exp\left[-(x^{2}+a^{2})/2\right]I_{m-1}\left(ax\right)$, m≥1
$L_{\nu }\left(\cdot \right)$	The generalized Laguerre function of order ν [30] (Equation (8.972/1)): Lν(z)=F11(−ν;1;z)
